# Transforming Two-Dimensional Carbon Allotropes into Three-Dimensional Ones through Topological Mapping: The Case of Biphenylene Carbon (Graphenylene)

**DOI:** 10.1021/acs.jpca.4c01339

**Published:** 2024-08-23

**Authors:** Raphael M. Tromer, Levi C. Felix, Ray H. Baughmann, Douglas S. Galvao, Cristiano F. Woellner

**Affiliations:** † Applied Physics Department, State University of Campinas, Campinas, SP 13083-970, Brazil; ‡ Center for Computational Engineering & Sciences - CCES, State University of Campinas, Campinas, SP 13083-970, Brazil; § Alan G. MacDiarmid NanoTech Institute, University of Texas at Dallas, Richardson, Texas 75080, United States; ∥ Physics Department, Federal University of Paraná - UFPR, Curitiba, PR 81531-980, Brazil

## Abstract

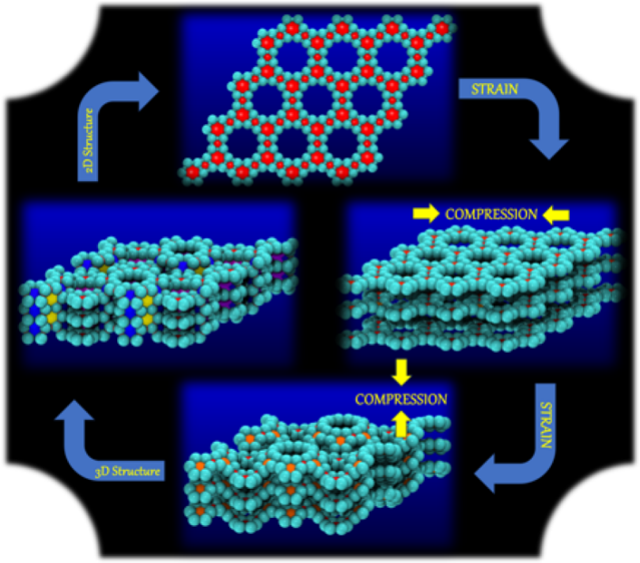

In this work, we propose a new methodology for obtaining three-dimensional (3D) carbon allotrope structures from 2D ones through topological mapping. The idea is to select a 3D target structure and “slice” it along different structural directions, creating a series of 2D structures. As a proof of concept, we chose the tubulane structure 12-hexa(3,3) as a target. Tubulanes are 3D carbon allotropes based on cross-linked carbon nanotubes. One of the obtained 2D “sliced” structures was mapped into the biphenylene carbon (BPC). We showed that compressing BPC in-plane, biaxially, followed by compression along the *z* direction using different strain rates could generate not only the target tubulane 12-hexa(3,3) structure but also at least two others: bcc-C6 and an unreported member of the tubulane family, which we called tubulane X. The methodology proposed here is entirely general; it can be used coupled with any quantum method. Considering that the 2D biphenylene carbon network, which is closely related to BPC, has been recently synthesized, the approach proposed here opens new perspectives to obtain new 3D carbon allotropes from 2D structures.

## 1 Introduction

The experimental realization of single-layer graphene^1^ created a revolution in materials science. Graphene is a two-dimensional (2D) carbon allotrope with unique electronic and mechanical properties that have been exploited in many applications.^2−6^

The advent of graphene renewed the interest in other 2D carbon allotrope materials and structures that were proposed before graphene, such as biphenylene networks (BPN)^7,8^ and graphynes,^9^ which were recently synthesized.^10,11^ It also stimulated the search for other noncarbon 2D materials.^12^ However, like graphene, most of these 2D materials are obtained from lamellar-like structures (the so-called van der Waals solids).^13^ Recently, the first 2D material from non-van der Waals solids, named hematene, was obtained from liquid exfoliation^14^ of 3D hematite. Following the same approach, several new 2D structures from 3D ones have been reported.^15^

A natural question is about the inverse process, i.e., how to obtain 3D structures from 2D ones. Of particular interest would be to obtain new 3D structures from 2D ones already experimentally realized. In fact, this is not a new idea. We have the case of diamond (a 3D structure) being obtained from graphite layers (2D structures) under high pressure and/or high temperature.^16−19^ However, topologically mapping 2D structures into 3D ones is not a trivial problem, and only a few examples are reported in the literature.^20,21^

In this work, we propose a new theoretical approach to obtaining 3D carbon allotropes from 2D ones. The main idea is to select a 3D target structure and structurally slice it along different crystallographic directions, creating a series of single-layer 2D structures. These selected 2D structures are then fully geometrically optimized and contrasted with existing or theoretically proposed 2D carbon allotropes. For the best candidates, we apply a mechanical chemistry-like process composed of a few steps: (i) creating a few-layer model of the chosen candidate structure; (ii) applying a biaxial, in-plane (*x* and *y*) compression at different strain rates to induce buckling in these 2D layers; and (iii) then repeating the process along the *z* direction until the layers chemically react, forming a 3D structure.

**Figure 1:**
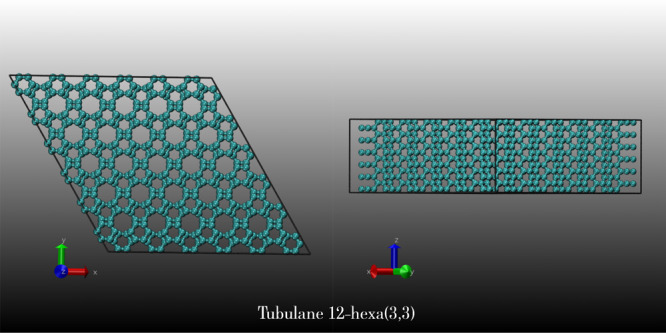
Left and right: the top and lateral views of the tubulane 12-hexa(3,3), the target structure.

As a proof of concept, we chose the tubulane structure 12-hexa(3,3) as a target (see Figure 1). Tubulanes are 3D carbon allotropes based on cross-linked carbon nanotubes.^22^ Their synthesis has remained elusive up to now.

**Figure 2:**
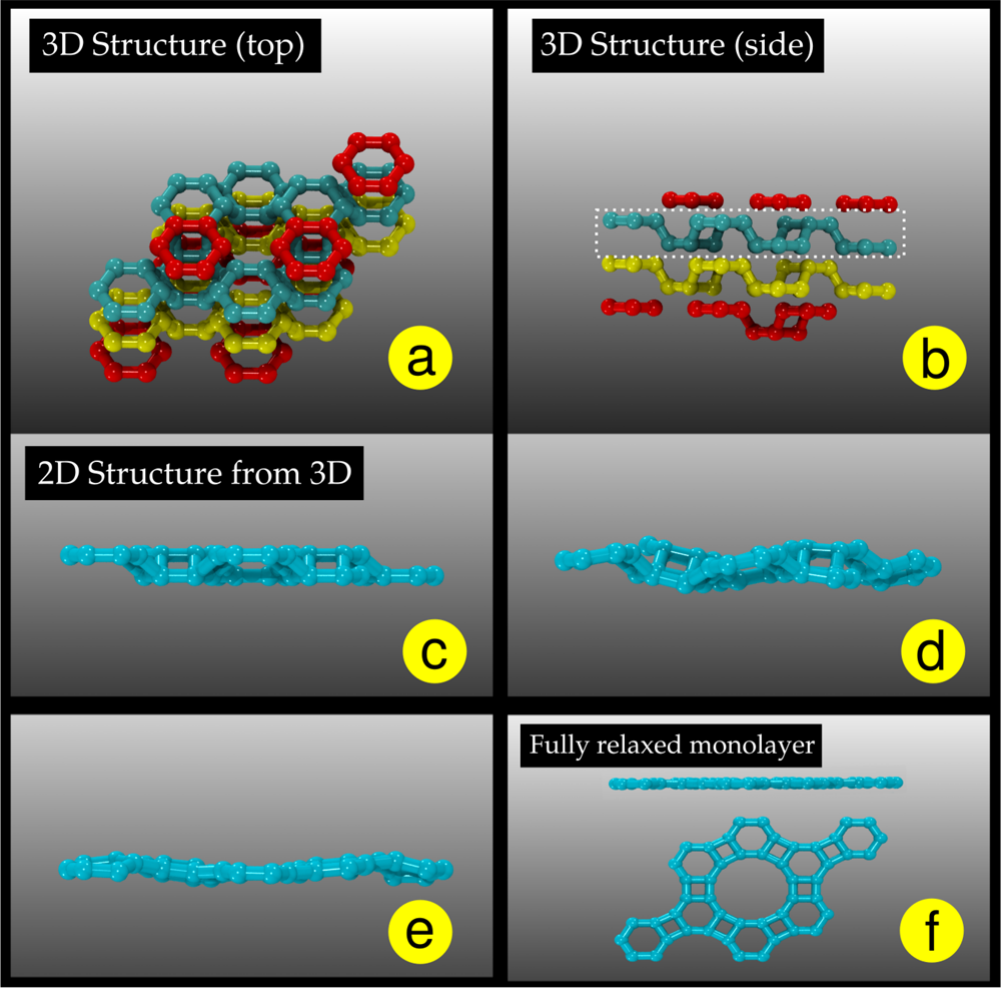
(a) Top view of the target structure, tubulane 12-hexa(3,3). For a better view, the interlayer bond is made transparent; (b) lateral view of (a) selected “sliced” layers. The dashed rectangle indicates one of the possible “sliced” 2D structures; (c–e) different stages of the geometry optimizations of panel (b); (f) lateral and top views of the optimized structure, identified as biphenylene carbon (BPC) (graphenylene).^9^

In Figure 2, we present the top (Figure 2a) and lateral views (Figure 2b) of a supercell of 12-hexa(3,3) containing three layers (indicated by different colors). In Figure 2c, we present one of the selected sliced 2D structures. Figure 2d–f shows representative snapshots of the optimization process (see the Materials and Methods section). In Figure 2f, we present the optimized structure (top and lateral views). Interestingly, this structure can be mapped into the biphenylene carbon (BPC), one of the structures of the biphenylene network family.^10^ BPC was proposed by Baughman and collaborators in 1987.^9^ We then analyzed the topological transformations that can generate 3D structures from 2D BPC.

## 2 Materials and Methods

**Figure 3:**
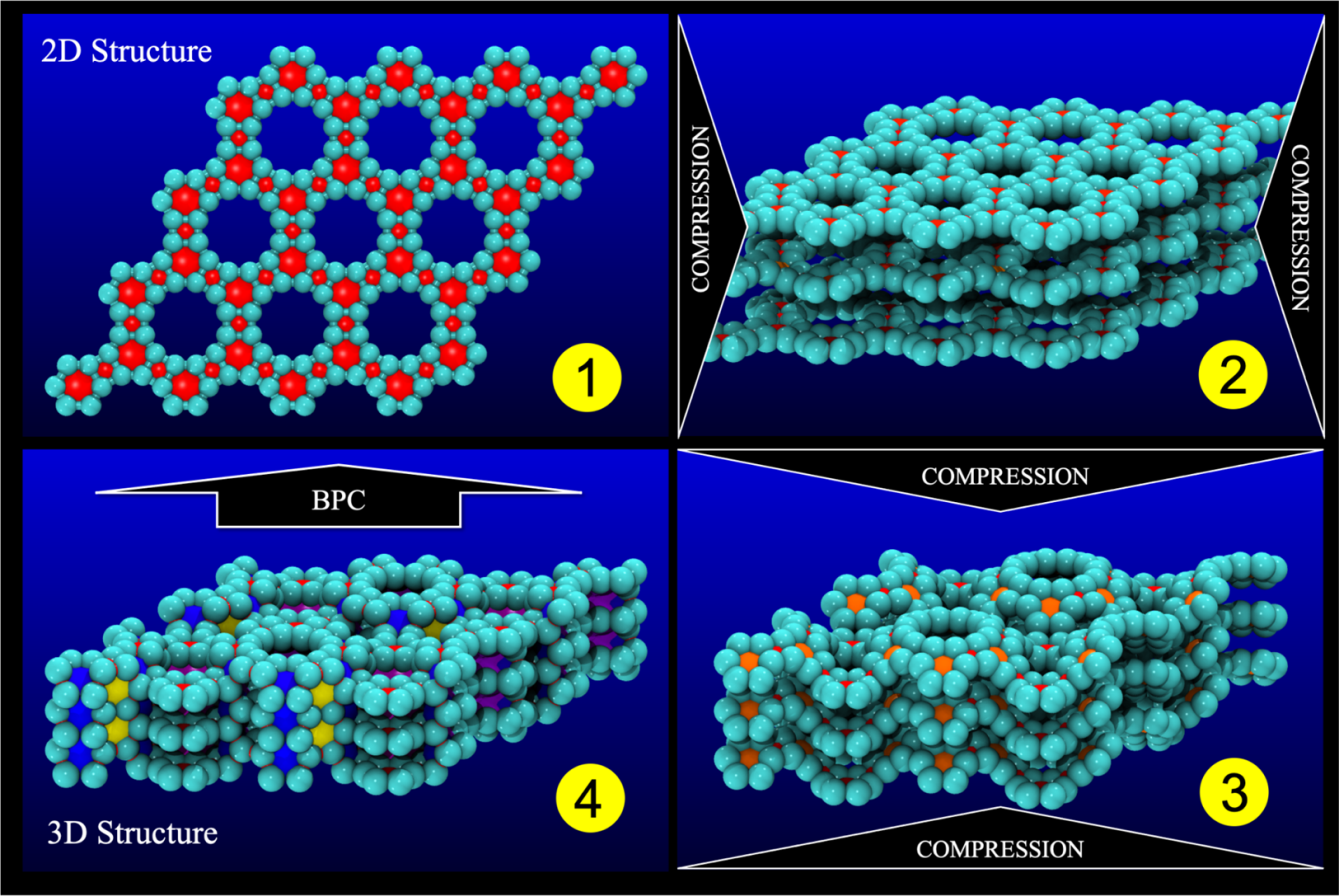
Schematic representation of how the 3D structures are obtained from 2D ones. The optimized BPC structure (1) is stacked (2) and compressed along the *x*, *y*, and *z* directions (2 and 3) until a stable 3D crystalline structure is obtained (4).

To obtain the 3D carbon allotrope structures from 2D BPC layers, we consider a mechanical chemistry-like process, following the three steps shown in Figure 3, and a more detailed scheme is presented in Figure S1 of the Supporting Information.

First, we consider a BPC supercell of three overlapping layers initially separated by 2 Å to prevent any covalent chemical bonds before geometry optimizations. All geometry optimizations were carried out using the semiempirical PM6-DH2 Hamiltonian (including van der Waals corrections), as implemented in the MOPAC2016 code.^23,24^ We chose a convergence criterion for geometry optimization when the gradient is less than 0.1. We stress that the initial minimum distances between the BPC layers are always large enough to avoid covalent bond formation among layers. This ensures that no bond formation occurs before the in-plane compression process takes place.

We want to remark that we chose to apply the compression first along *xy* and then along the *z* direction and not the opposite because the compression along the *xy* direction induces a buckling in the 2D structures, thus increasing their reactivity. This provides many degrees of freedom for the formation of the target 3D structure. The compression could also be applied first along the *z* direction, which keeps the atoms in the plane. Although the formation of 3D structures could be possible, it is less effective than first applying compression along *xy*.

Once the system is geometrically optimized, we apply a biaxial (simultaneously) strain along the *x* and *y* directions until the layers chemically react, forming a 3D structure. The compression value/rate can be arbitrarily chosen, corresponding to steps 2 and 3 in Figure 3. Different biaxial strain values (initial conditions) applied to 2D layers can, in principle, lead to different 3D structures. After the compression along the *x* and *y* direction is completed, the compression along the *z* direction is also applied (step 3 in Figure 3). Before this compression, the system is geometrically relaxed along the *z* direction.

As the layers are compressed, they deform and can react, forming intralayer and interlayer covalent bonds (this can occur in steps 2 and 3). Once a well-defined 3D structure is formed (step 4 in Figure 3), the compression process is stopped and the obtained 3D structure is then fully optimized (lattice vectors and atom positions, with no constraints). If the obtained 3D structure is not a defined target, then the process can be repeated using different values of the applied strain values and rate compression.

## 3 Results and Discussion

**Figure 4:**
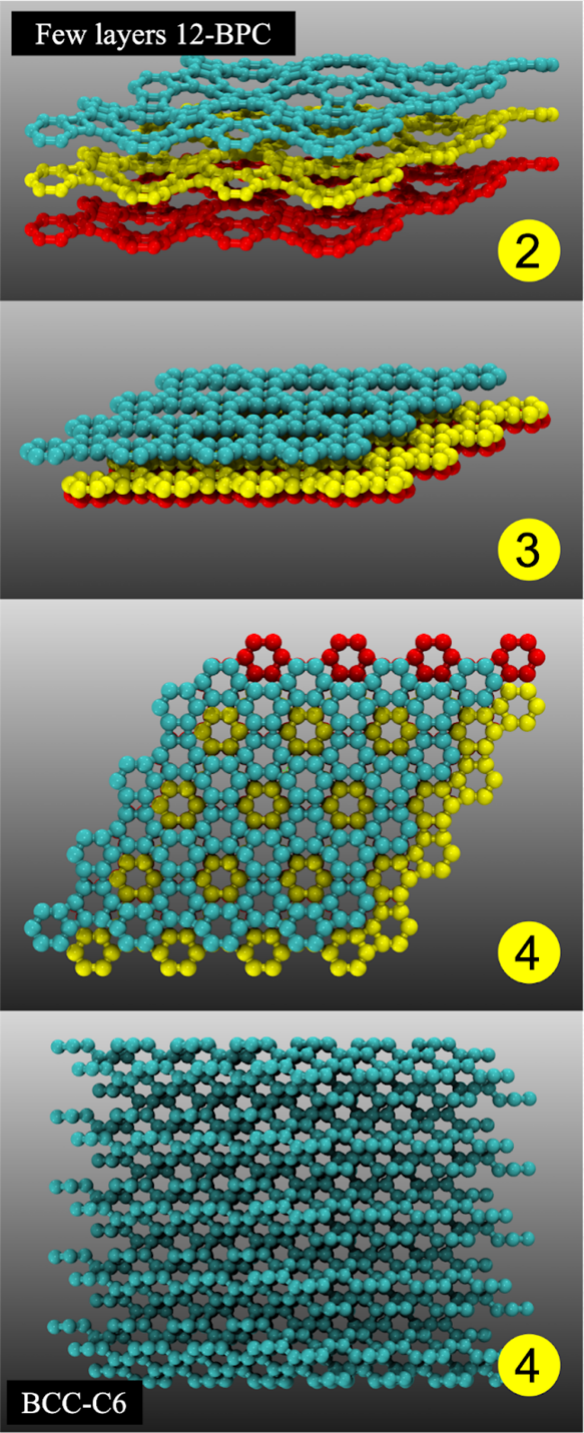
Representative snapshots of the structural stages from 2D to 3D structures. Compressed corrugated (buckled) stacked BPC (2). The layers slide and become less buckled (3). Top and lateral views of the obtained 3D structure identified as bcc-C6.^21^

Following the steps shown in Figure 3, we used a biaxial strain of 2% along the *xy* plane followed by a compression of 10% along the *z* direction (in steps of 0.5 Å); we then observed the formation of a well-defined 3D structure. During the structural transformations, the number of atoms is kept constant; we considered 144 carbon atoms in the supercell for all cases discussed in this work. When the system is compressed along the *z* direction, the layers are displaced along plane *xy*, breaking the initial AA coupling, as shown in step 3 from Figure 4. In Figure 4, we present a series of representative snapshots of the process. The analysis of the structure (see Table 1) showed that the obtained 3D structure was not the expected tubulane target but a well-known carbon allotrope known as bcc-C6.^25^ The MOPAC prediction for the formation energy of the resultant 3D structure (bcc-C6) is −8.7 eV/atom, which is the same value from DFT calculations.^25^ For the bcc-C6 case, we conducted a detailed analysis of the transformation process from 2D layers to the 3D system. This transition is illustrated in Figure S2, which is accompanied by a brief discussion of the process.

**Table 1:** Summary of the Structural Information of the Obtained 3D Structures and Their Parent 2D BPC

**structures**	**atoms**	**space group**	**unit cell optimized lattice parameters**
BPC^27^	48	*P*6/*mmm*(191)	*a* = *b* = 6.68
			*c =* 20.00
			α = β = 90
			γ = 60
bcc-C6^25^	144	*P*3̅*m*1 (164)	*a* = *b* = 13.10
			*c =*6.70
			α = β = 90
			γ = 60
tubulane 12-hexa(3,3)^22^	144	*P*63/*mmc* (194)	*a* = *b* = 12.09
			*c =* 7.79
			α = β = 90
			γ = 60
tubulane X (this work)	144	*P*6/*mmm* (191)	*a* = *b* = 12.70
			*c* = 7.90
			α = β = 90
			γ = 60

**Figure 5:**
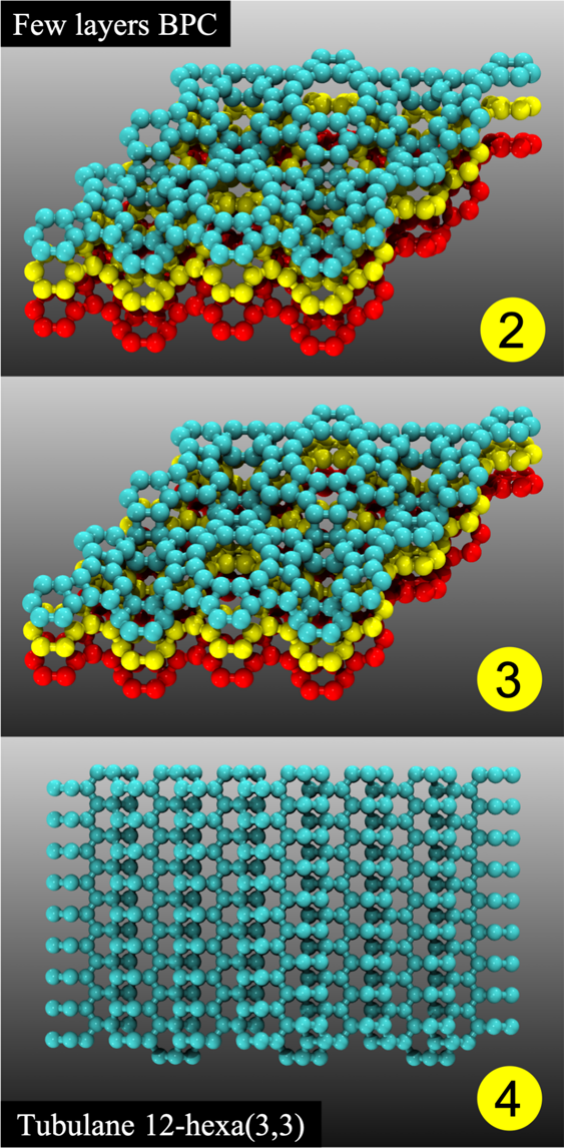
Representative snapshots of the structural stages from 2D to 3D ones. Compressed corrugated (buckled) stacked BPC (2). Top (3) and lateral view (4) of the obtained 3D structure, identified as the target tubulane 12-hexa(3,3).^22^

As the obtained 3D structure was not the defined target, we then repeated the process, considering the same initial conditions but increasing the compression rate along the *xy* plane. After the system converges to an applied compression of 2% along the *xy* plane (see step 2 from Figure 2), it is compressed again by 2%, and the process is repeated until the total compression is 10%, as shown in step 2 of Figure 5. The layer curvature now is larger than in the previous case, which will be reflected in different chemical reactivities.

The system is then compressed by 10% along the *z* direction in steps of 0.5 Å (step 3 from Figure 5). Again, a well-formed 3D structure is obtained. The crystallographic analysis (see Table 1) shows that it is the desired target tubulane 12-hexa(3,3)^22^ (see step 4 from Figure 5). Unaware of the tubulane work, some authors “rediscovered” this structure years later and named it bct.^26^

Another parameter that affects the resulting 3D structure is the compression rate along the *z* direction. For different rate values, different 3D systems are obtained. In Figure S3 of the Supporting Information, we present the results for the structure obtained using the same procedure to obtain the tubulane 12-hexa(3,3) but changing the *z*-compression rate to a step of 0.8 Å. We could not identify this 3D structure from the literature, but it is extremely similar (see Table 1) to the structures of the tubulane family, but not one of the structures listed in the original tubulane paper; we named it tubulane X. To test the structural stability of tubulane X, we carried out the phonon spectra using the well-known Tersoff potential.^28^ No negative frequencies were observed, suggesting that the structure is stable. To test the stability at high temperatures further, we also carried out ab initio molecular dynamics simulations using the SIESTA code^29^ for 4 ps at 1000 K and using an NVT ensemble. No significant structural changes were observed, further confirming the structural stability. The coordinates of optimized tubulane X are available upon request.

**Figure 6:**
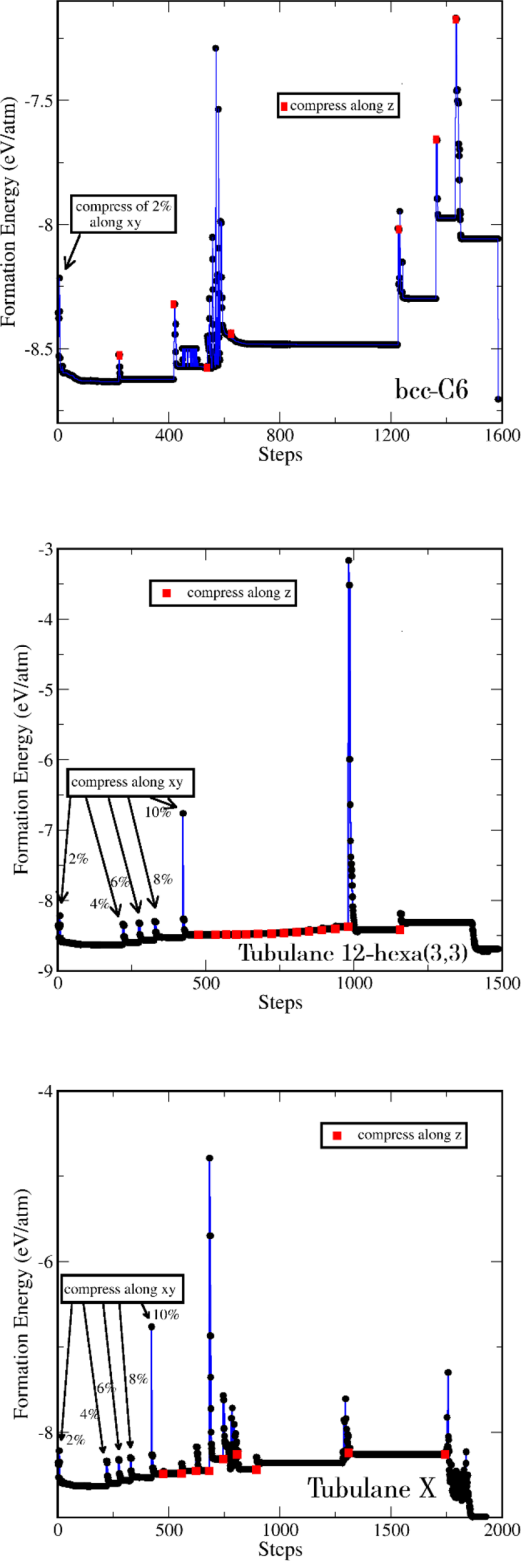
Energy profiles during the structural transformation processes from 2D to 3D structures. Top to bottom, bcc-C6, tubulane 12-hexa(3,3), and tubulane X. As can be seen from this figure, although some of the intermediate structural transformations generate 3D structures with higher energy (indicated by the peaks) than the 2D ones, all final optimized 3D structures have lower energy values.

In Figure 6, we present the formation energy as a function of the simulation steps for the above-discussed cases. Initially, we have the compression along the *xy* plane followed by successive compressions along the *z* direction (red squares). From this figure, we can see that for all cases, the obtained 3D structures are more stable than their “parent” 2D ones. The whole process can be better understood from Videos S1–S3 in the Supporting Information.

For the bcc-C6 case, we have analyzed in detail the transformation that occurs during the transition process from 2D layers to the 3D system, as shown in Figure 6a, which occurs with the energy profile. We plotted the radial distribution function to investigate the transition process considering the range from 0 to 3 Å. We see that for the iterative process up to 420 steps, the layers still interact only through van der Waals forces and are perfectly aligned in the AAA configuration, with the middle layer showing higher roughness than the two outer layers. The radial function shows three peaks around 1.5 Å, representing the different types of bonds that are slightly different. Another relevant peak is observed around 2.5 Å, representing the interlayer interaction.

**Figure 7:**
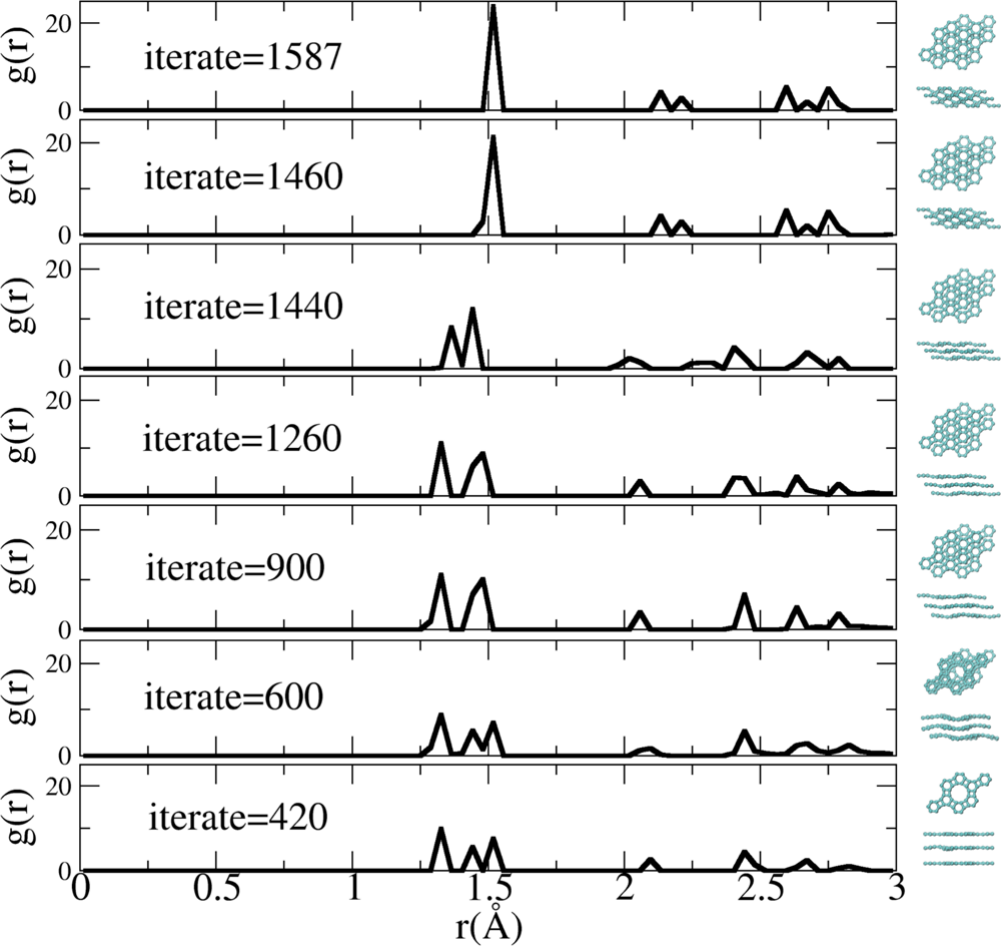
Radial distribution function *g*(*r*) for the bcc-C6 structure.

At 600 iterations, the radial function remains practically unchanged except that the layers begin to shift within the plane, starting a misalignment. By 900 iterations, the layers are completely misaligned, forming an ABC configuration. Additionally, we observe only two peaks around 1.5 Å, corresponding to the bonds between atoms located within the same layer. This behavior continues until 1440 iterations, where only the distance between layers changes due to compression along the *z* axis. Shortly after, around 1460 iterations, the system transitioned to the 3D structure, where the layers are bonded. The radial distribution function shows only one peak at around 1.5 Å, representing the bond length between carbon atoms (Figure 7). We also note that the peak near 2.5 Å disappeared because the layers are now bonded together. An additional step is performed, regarding optimizing the atoms and lattice vectors.

Up to now, we have discussed the procedures to transform the 2D BPC into (at least) three different 3D structures. Further validation of these topological transformations is to carry out the inverse process, i.e., “slicing” bcc-C6, tubulane 12-hexa(3,3), and tubulane X, to recover the 2D BPC structure. We carried out this process, and the obtained sliced 2D structures were then geometrically optimized. As expected, the 2D BPC structure is reobtained (see Figures S4–S6 in the Supporting Information), thus further validating our topological approach.

Also to be considered are the recent advances in mechanochemistry^30^ that make our proposed approach closer to reality. Mechanochemistry is a new fascinating field that explores chemical reactions induced by mechanical force rather than more traditional methods like wet chemistry, heat, or light.^30^ By grinding, milling, or shearing materials together, mechanochemistry can unlock unique reaction pathways.^31^ These innovative approaches offer advantages regarding energy efficiency, reaction selectivity, and access to novel compounds. This also offers new pathways for better and more efficient green chemistry. Mechanochemistry has applications across various fields from materials science to pharmaceuticals. The recent advances^32^ open new perspectives for materials science. We hope that the present work will further stimulate other studies along these lines.

## 4 Summary and Conclusions

In this work, we propose a new methodology for obtaining 3D carbon allotrope structures from 2D ones through topological mapping. The idea is to select a 3D target structure and “slice” it along different structural directions, creating a series of 2D structures. These 2D structures are then fully geometrically optimized (we used the quantum Hamiltonian PM6-DH2, as available in the MOPAC code^23^) and topologically mapped into existing or theoretically proposed 2D carbon allotropes. As proof of concept, we chose the tubulane structure 12-hexa(3,3) as a target. Tubulanes are 3D carbon allotropes based on cross-linked carbon nanotubes.^22^ One of the obtained 2D “sliced” structures was mapped into the biphenylene carbon (BPC). Initially, the BPC compression process did not yield the target structure but the bcc-C6. Then, using different parameters, the target structure (tubulane 12-hexa(3,3)) was obtained as well as an unreported member of the tubulane family, which we called tubulane X. For completeness, we carried out the “reverse test”, “slicing” again the obtained 3D structures (bcc-C6, tubulane 12-hexa(3,3), and tubulane X), and their “parent” 2D BPC was reobtained for all cases (see Figures S4–S6 in the Supporting Information). The methodology proposed here is completely general, and it can be used coupled with any quantum method. Considering that new 2D carbon allotropes, such as the biphenylene carbon network, which is closely related to BPC, have been recently synthesized (as well as other related structures, such as graphynes and 2D fullerene networks), the approach proposed here opens new perspectives to obtain new 3D carbon allotropes from 2D structures.

## Supporting Information Available

Mechanochemical synthesis (BPC-12 to bcc-C6) (MP4)
Mechanochemical synthesis (BPC-12 to tubulane 12-hexa(3,3)) (MP4)
Mechanochemical synthesis (BPC-12 to tubulane X) (MP4)
Schematics of the 3D structures obtained from 2D ones and vice versa, including the energy profiles during the structural transformation processes from 2D to 3D structures (PDF)
