# Supplementary material for: Transforming Two-Dimensional Carbon Allotropes into Three-Dimensional Ones through Topological Mapping: The Case of Biphenylene Carbon (Graphenylene)
Source: J Phys Chem A. 2024 Aug 23;128(35):7346–52. doi: 10.1021/acs.jpca.4c01339 (PMC11382271; doi:10.1021/acs.jpca.4c01339)
Supplement: Supplementary file 4 — jp4c01339_si_004.pdf [file jp4c01339_si_004.pdf]

Transforming 2D carbon allotropes into 3D ones through topological mapping: The case of biphenylene carbon (graphenylene)

Raphael M. Tromer[1,2], Levi C. Felix[1,2], Ray H. Baughmann[3], Douglas S. Galvao[1,2], and Cristiano F. Woellner[4*]

[1]Applied Physics Department, State University of Campinas, Campinas/SP, 13083-970, Brazil

[2]Center for Computational Engineering & Sciences - CCES, State University of Campinas, Campinas/SP, 13083-970, Brazil.

[3]Alan G. MacDiarmid NanoTech Institute, University of Texas at Dallas, Richardson, TX, 75080 USA

[4]Physics Department, Federal University of Paraná - UFPR, Curitiba/PR, 81531-980, Brazil.

[*]Corresponding author. E-mail address: woellner@ufpr.br

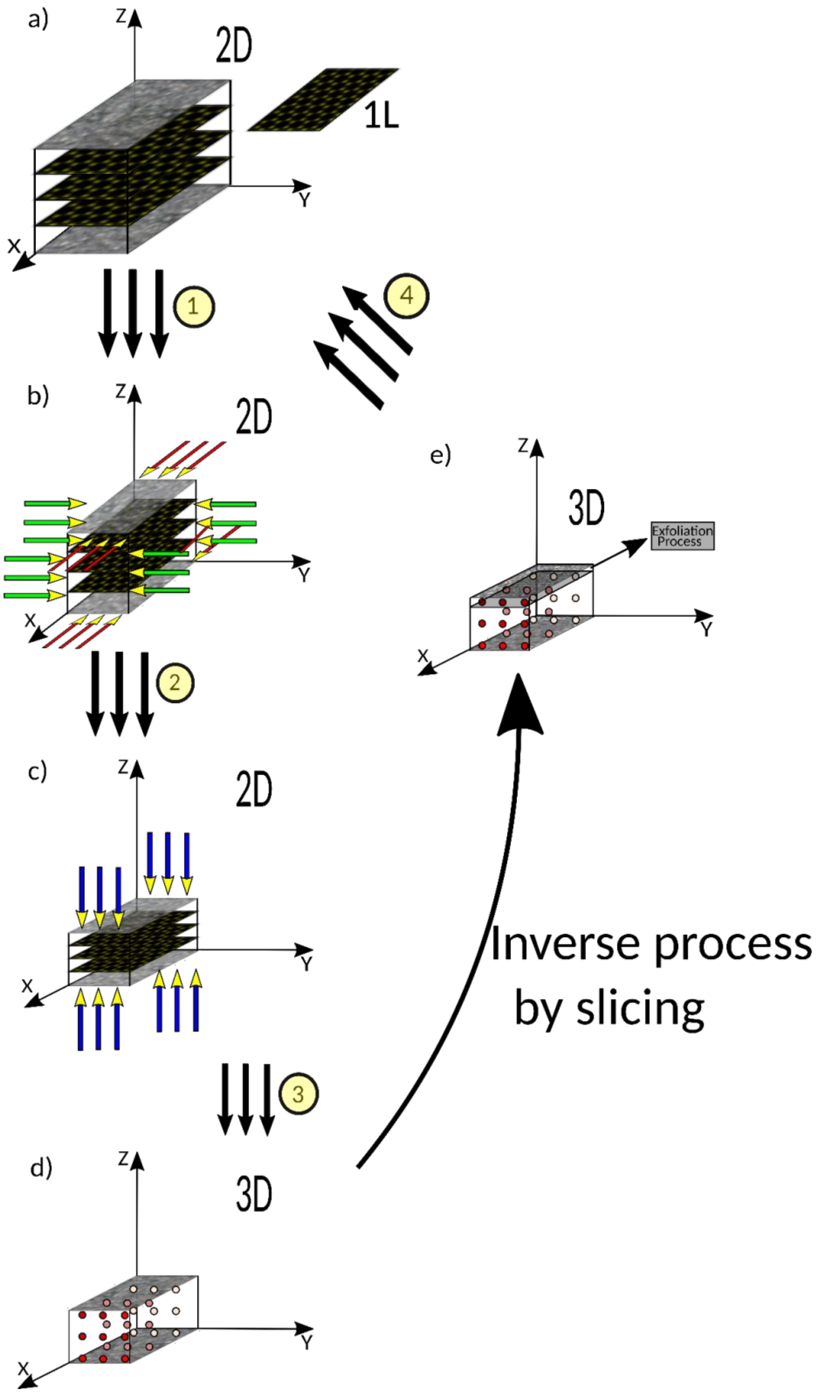

a)
2D
1L
1
4
b)
2D
e)
3D
Exfoliation Process
2
c)
2D
Inverse process
by slicing
3
d)
3D

Figure S1. Scheme of obtaining 3D structures from 2D ones. (1) 'Sliced' 2D layers from the 3D'target' structure are compressed in different directions (2) until a stable 3D structure is obtained (3). To test for completeness, the 'inverse' process is carried out, 'slicing' the obtained 3D structure to obtain new 2D ones (4). If the obtained 3D structure is not the desired 'target' the process is repeated using different compressing/slicing parameters/2D structures.

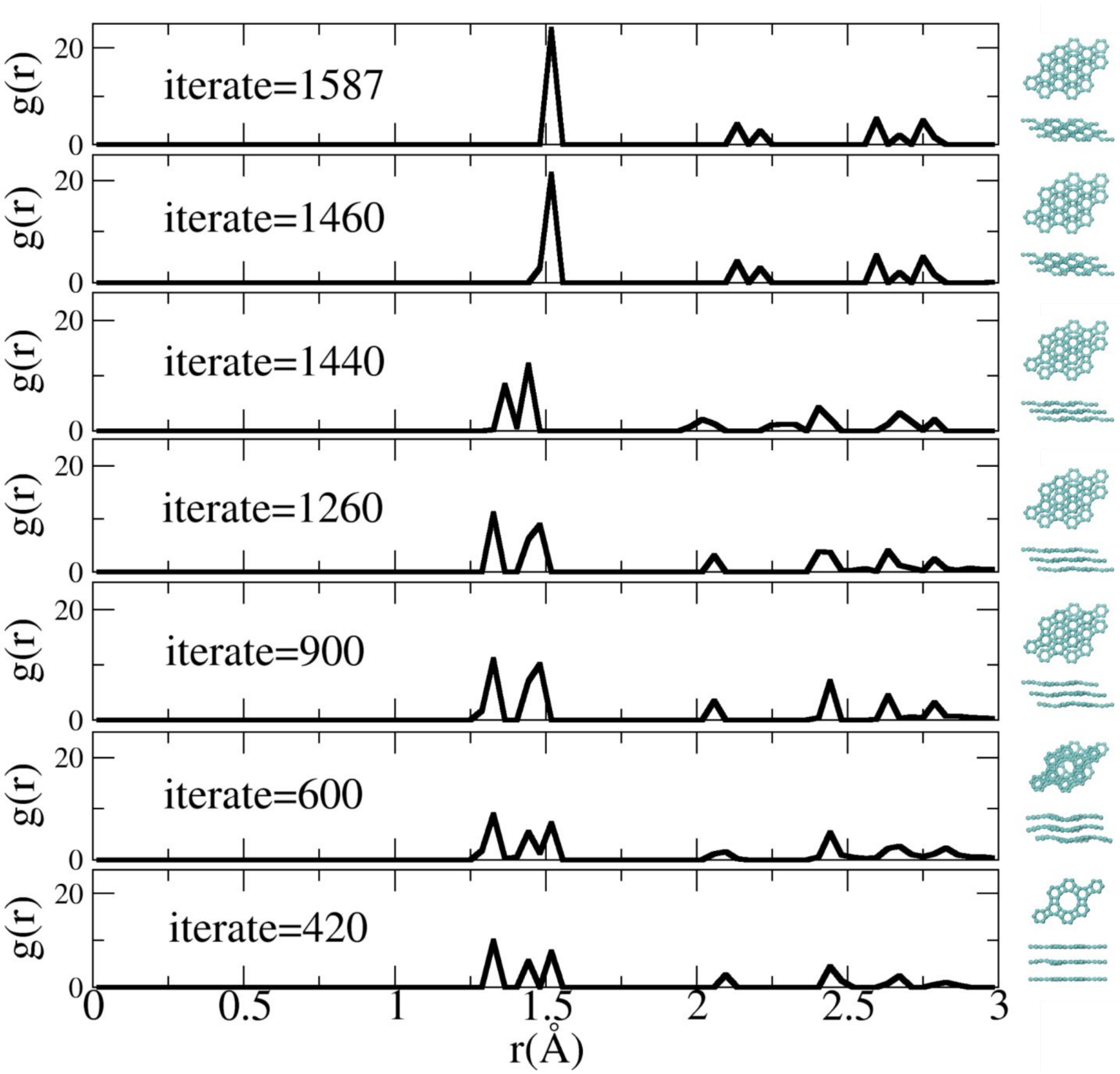

Figure S2. Radial function distribution for the bcc-C6 case.

For the bcc-C6 case, we analyzed in detail the transformation that occurs during the transition process from 2D layers to the 3D system, as shown in Figure 6-a), which occurs with the energy profile. To investigate the transition process, we plotted the radial distribution function considering the range from 0 to 3 Å. We see that for the iterative process up to 420 steps, the layers are still interacting only through van der Waals forces and are perfectly aligned in the AAA configuration,

with the middle layer showing greater roughness compared to the two outer layers. The radial function shows three peaks around 1.5 Å, representing the different types of bonds that are slightly different. Another relevant peak is observed around 2.5 Å, representing the inter-layer interaction.

At 600 iterations, the radial function remains practically unchanged, except that the layers begin to shift within the plane, initiating a misalignment. By 900 iterations, the layers are completely misaligned, forming an ABC configuration. Additionally, we observe only two peaks around 1.5 Å, corresponding to the bonds between atoms located within the same layer. This behavior continues until 1440 iterations, where only the distance between layers changes due to compression along the Z-axis. Shortly after, around 1460 iterations, we observe that the system has transitioned to the 3D structure where the layers are bonded. The radial distribution function shows only one peak around 1.5 Å, representing the bond length between carbon atoms. We also note that the peak near 2.5 Å has disappeared because the layers are now bonded together. Remember that an additional step is performed regarding the optimization of the atoms and lattice vectors.

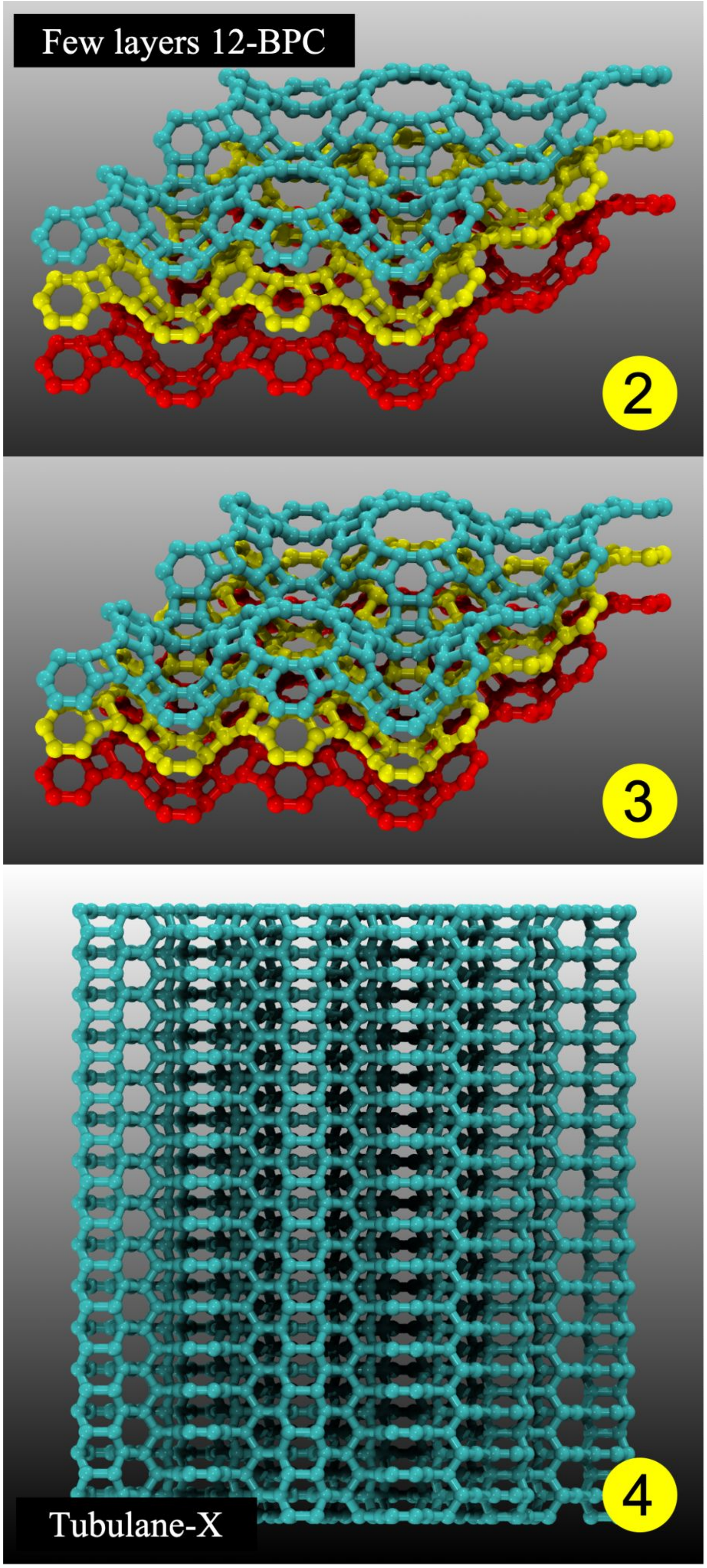
Few layers 12-BPC
2
3
Tubulane-X
4

Figure S3. Representative snapshots of the structural stages from 2D to 3D ones. Compressed corrugated (buckled) stacked BPC (2). The top (3) and lateral view (4) of the obtained 3D structure, named tubulane X, which is an unreported new member of the tubulane family [22].

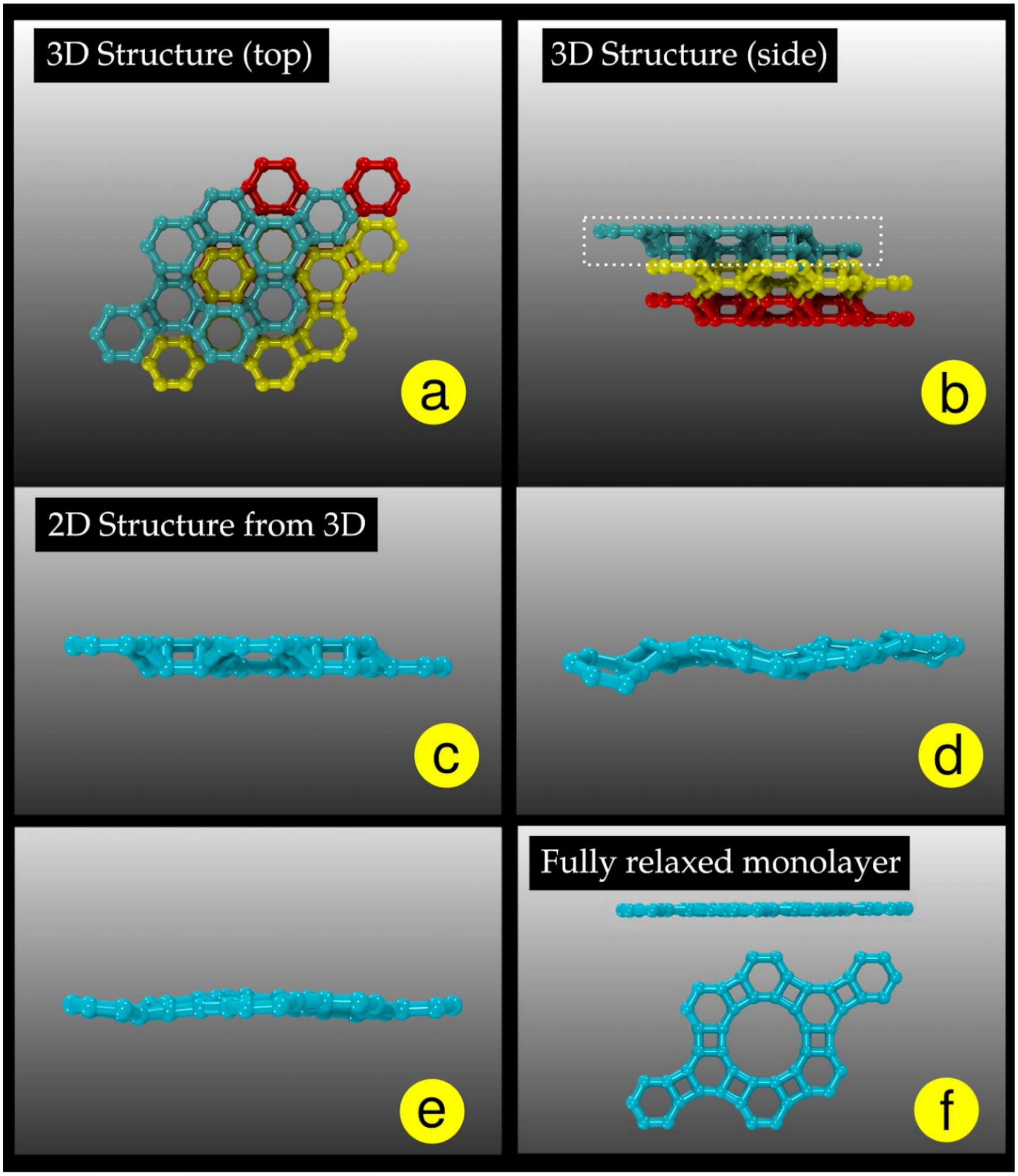

Figure S4: 'Reverse' test '. 'Sliced' structures form the obtained 3D bcc-C6 [21] crystal recover the 'parent' 2D BPC.

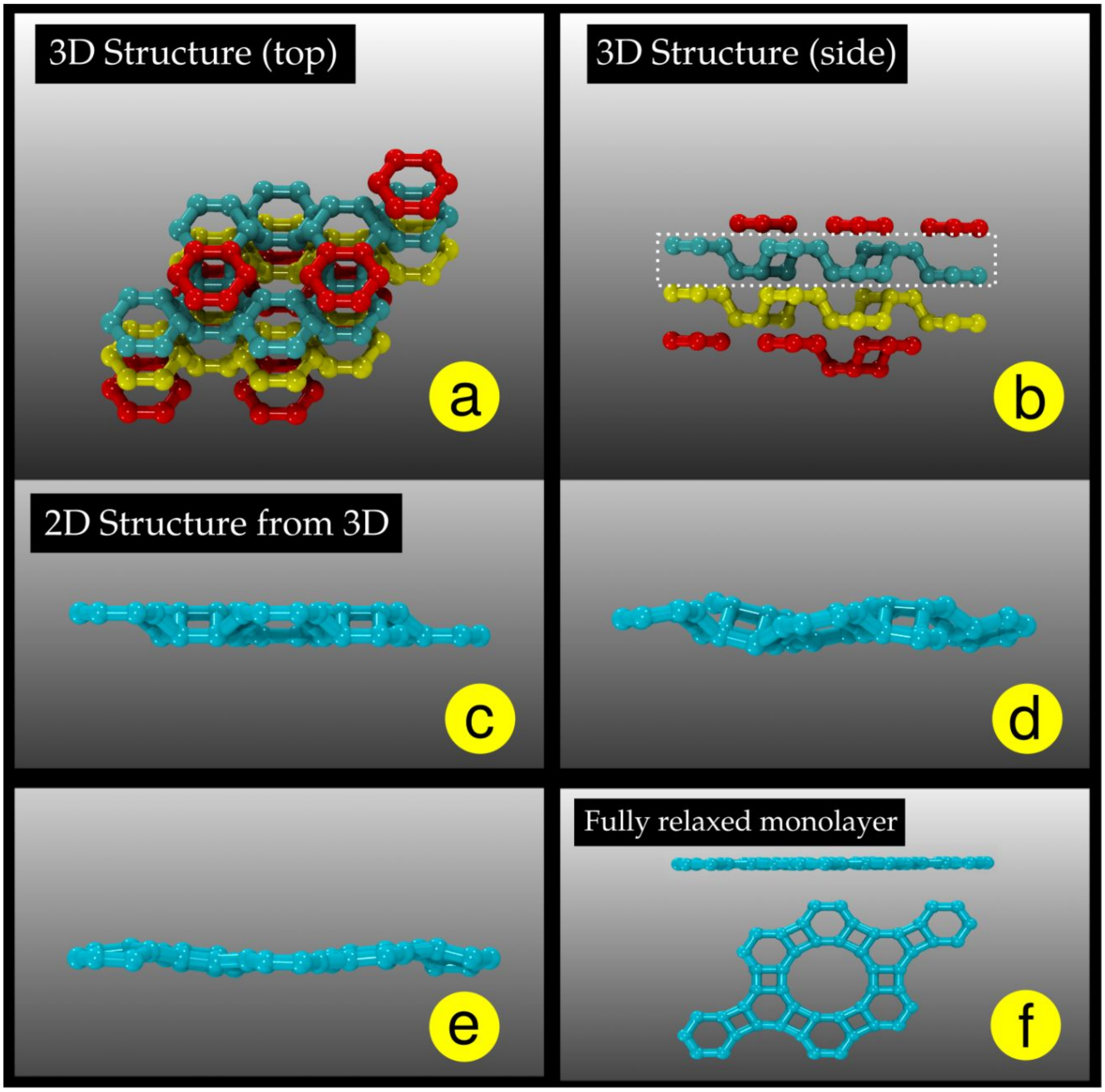

Figure S5: 'Reverse' test '. 'Sliced' structures form the obtained 3D tubulane 12-hexa(3,3) [22] recover the 'parent' 2D BPC.

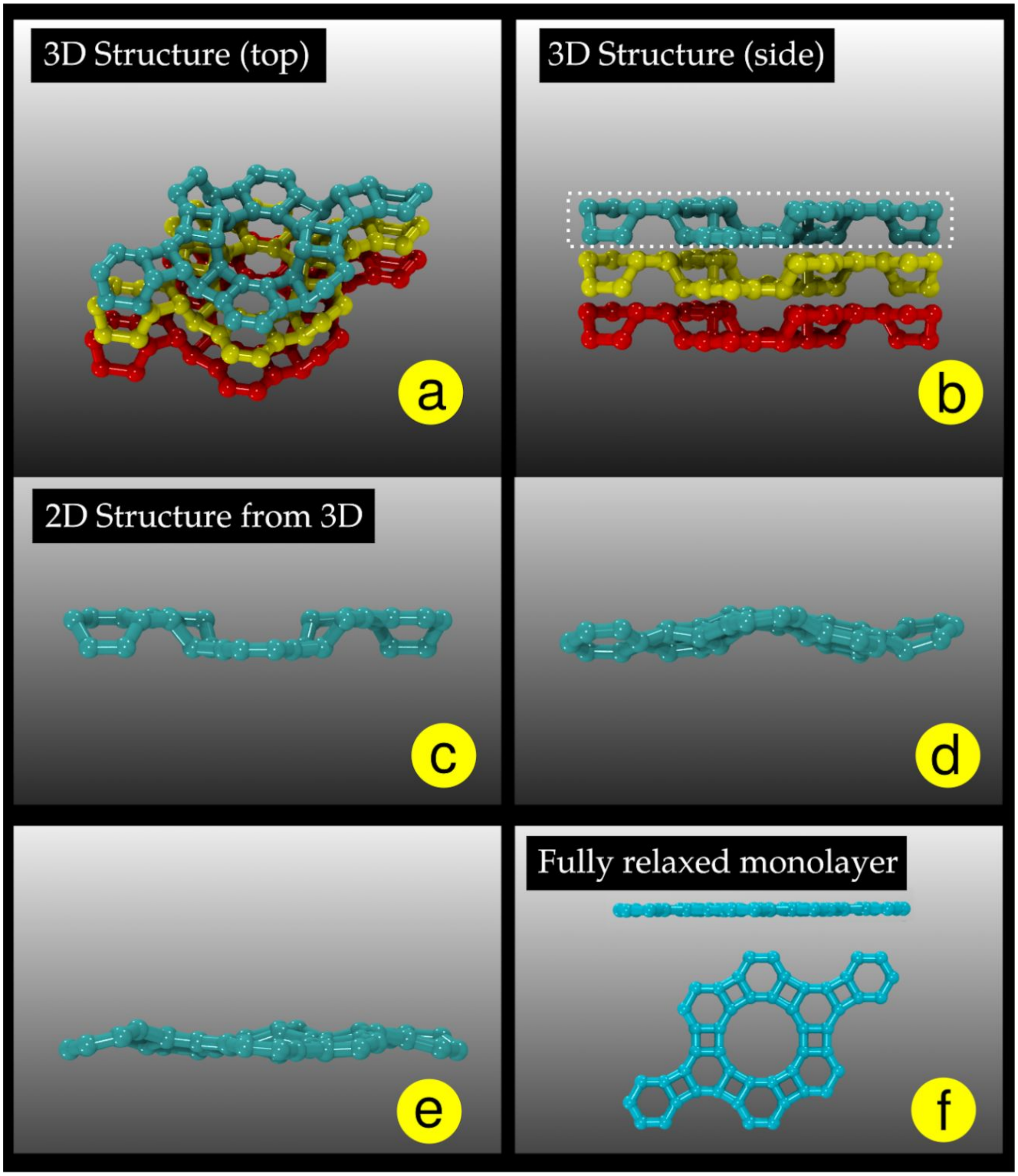

Figure S6: 'Reverse' test '. 'Sliced' structures from the obtained tubulane X crystal recover the 'parent' 2D BPC.
